# In-Orbit Calibration of Phase Retardance for Channeled Spectropolarimeter

**DOI:** 10.3390/s23052642

**Published:** 2023-02-28

**Authors:** Youzhi Dong, Xueping Ju, Jing Yuan, Changxiang Yan, Tao Zhang

**Affiliations:** 1 Changchun Institute of Optics, Fine Mechanics and Physics, Chinese Academy of Sciences, Changchun 130033, China; 2 University of the Chinese Academy of Sciences, Beijing 100049, China; 3 Center of Materials Science and Optoelectrics Engineering, University of Chinese Academy of Sciences, Beijing 100049, China

**Keywords:** calibration, channeled spectropolarimeter, phase retardance, high-precision

## Abstract

The phase retardance of the optical system (PROS) is a crucial factor limiting the accuracy of the Stokes vector reconstruction for the channeled spectropolarimeter. The dependence on reference light with a specific angle of polarization (AOP) and the sensitivity to environmental disturbance brings challenges to the in-orbit calibration of PROS. In this work, we propose an instant calibration scheme with a simple program. A function with a monitoring role is constructed to precisely acquire a reference beam with a specific AOP. Combined with numerical analysis, high-precision calibration without the onboard calibrator is realized. The simulation and experiments prove the effectiveness and anti-interference characteristics of the scheme. Our research under the framework of fieldable channeled spectropolarimeter shows that the reconstruction accuracy of $S_{2}$ and $S_{3}$ in the whole wavenumber domain are 7.2 × 10^−3^ and 3.3 × 10^−3^, respectively. The highlight of the scheme is to simplify the calibration program and ensure that the PROS high-precision calibration is not disturbed by the orbital environment.

## 1. Introduction

Polarimetric spectral imaging is a powerful tool with various applications [1], such as atmospheric aerosol characterization [2,3] and material analysis [4,5,6,7]. Channeled spectropolarimetry, proposed by Oka et al. [8,9], enables full Stokes vector acquisition with simple optical structure and fixed polarization components. All these advantages promote the development of channeled spectropolarimeters based on different structures [10,11]. Most of the reported schemes follow the assumption that the polarization aberrations of the optical systems have negligible impact on the measurement process and reconstruction by default. However, this idealized simplification is a concrete source of error in the context of large fields of view and the complexity of optical film design [12]. The phase retardance of the optical system (PROS) is a crucial factor. Specifically, the PROS characterizes the maximum phase difference in the eigenpolarization state. Due to the difference, the element produces different phase changes for beams with different polarization states. This effect is superimposed along the beam propagation path, limiting the accuracy of the reconstructed Stokes vectors. The PROS is quite sensitive to environmental disturbance [13]. Considering that the orbital environment will be affected by uncertainties such as temperature, launch vibration, and satellite attitude, in-orbit instant calibration of PROS is essential and meaningful.

So far, the quantitative analysis of PROS for the channeled spectropolarimeter has focused on the laboratory stage, requiring the assistance of a reference beam with a known polarization state. Stephen et al. [14] characterized the non-ideal factors of the system using linear operators to record the model response of the system by inputting reference beams with different angles of polarization (AOP). The corresponding data acquisition and processing are redundant. Yang, Xing et al. [15,16] simplified the process and considered the issue of polarization effects with radiation coupling. Linearly polarized beams at 22.5° and 45° were used for the quantitative calibration of PROS in the laboratory. However, it is challenging to achieve quantitative calibration of PROS in orbit. One factor is the dependence of the high-precision calibration on the reference beam. In existing in-orbit polarization calibration methods, the onboard calibrator with a built-in standard illuminant is the most effective way to acquire a specific reference beam. The supporting mechanical structure and control system adds to the bulk of the device and gets in the way of a compact implementation. The complexity of the components is also detrimental to long-term in-orbit maintenance. Another factor is that the onboard calibrator is independent of the observing system and can only achieve periodic calibrations. Environmental disturbances are out-of-synchrony for calibrations of different parameters.

In this work, we propose a novel scheme for the in-orbit calibration of the PROS for the channeled spectropolarimeter. A dynamic function with a monitoring role is constructed to precisely acquire a reference beam with a specific AOP at high frequencies to achieve the high-precision calibration of PROS without additional calibrators.

## 2. Modeling and Methods

### 2.1. Polarization Radiometric Calibration Model of Channeled Spectropolarimeter

**Figure 1:**
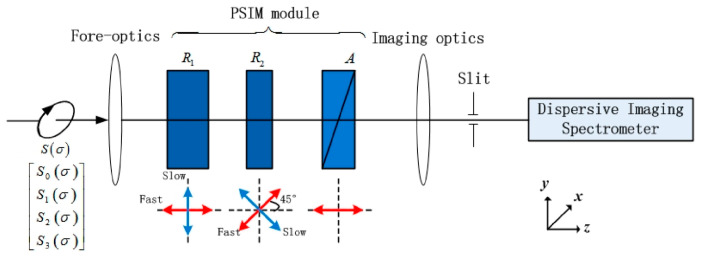
Optical schematic of the fieldable channeled spectropolarimeter.

The optical schematic of the fieldable channeled spectropolarimeter designed for airborne remote sensing is depicted in Figure 1 [16]. A polychromatic beam is modulated by the polarimetric-spectral intensity modulation (P-SIM) [8] after the collection and collimation of the fore-optics. The P-SIM consists of high-order retarders $R_{1}$, $R_{2}$ and a polarizer A. A dispersive imaging spectrometer receives the modulation spectrum. The phase retardance of the fore-optics $\delta_{fore}$ is analyzed as the only PROS source for this structure.

The crux of the calibration of $\delta_{fore}$ is the in-orbit acquisition of the reference beam with a specific AOP. We chose to “capture” the reference beam rather than “create” it. In the framework of the polarization radiometric transmission model, the corresponding relationship between the grayscale value of the spectrometer and the incident pupil radiance is expressed as:

(1) $$DN_{orbit}=A^{k}\cdot B_{orbit}+C,$$

where $DN_{orbit}$ is the original digital number DN; $A^{k}$ and C are the radiometric calibration coefficient and the DN of dark current, both of which can be accurately calibrated prior to orbit by mature absolute radiometric calibration. Furthermore, $B_{orbit}$ is the modulation spectrum radiance, characterized by [12,13,17]:

(2) $$B_{orbit}=nA\cdot M_{spec}\cdot M_{imag}\cdot M_{PSIM}\cdot M_{fore}\cdot S_{in-orbit},$$

where nA, linearly related to $A^{k}$, is the constant of the system effective gain coefficient; $M_{fore}$, $M_{PSIM}$, $M_{imag}$, and $M_{spec}$, correspond to the cascaded Muller matrix of four subsystems: fore-optics, P-SIM, imaging optics, and spectrometer, respectively. $S_{in-orbit}={\left[S_{0},S_{1},S_{2},S_{3}\right]}^{T}$ is the Stokes vector of the incident pupil beam. 

### 2.2. Methods for In-Orbit Calibration of the PROS

Here, we set some new parameters for the intuitive demonstration of derivation [16]:

(3) $$\left\{\begin{array}{l} a_{1,orbit}=\sin2\varepsilon_{1}\\ a_{2,orbit}=\cos2\varepsilon_{1}\\ b_{1,orbit}=\sin2\varepsilon_{2}\\ b_{2,orbit}=\cos2\varepsilon_{2}\\ c_{1,orbit}=\sin2\left(\varepsilon_{2}-\varepsilon_{1}\right)\\ c_{2,orbit}=\cos2\left(\varepsilon_{2}-\varepsilon_{1}\right) \end{array}\right.,\left\{\begin{array}{l} \;X_{0}=S_{0}+D_{fore-orbit}S_{1}\\ \;X_{1}=D_{fore-orbit}S_{0}+S_{1}\\ \;X_{2}=S_{2}+\delta_{fore-orbit}S_{3}\\ \;X_{3}=S_{3}-\delta_{fore-orbit}S_{2}\\ X_{12}=a_{2,orbit}X_{1}+a_{1,orbit}X_{2}\\ X_{123}=a_{1,orbit}X_{1}-a_{2,orbit}X_{2}+iX_{3} \end{array}\right..$$

It should be noted that: $\varepsilon_{1}$ and $\varepsilon_{2}$ characterize the angular difference between the actual setting position and the preset position of $R_{1}$ and $R_{2}$. In current mainstream cognition, researchers tend to treat them as error sources. In practice, however, these two parameters are deeply involved in the decoupling process, which will directly impact calibration accuracy. The following work will prove that they cannot simply be set to small quantities. $D_{fore-orbit}$ and $\delta_{fore-orbit}$ correspond to the diattenuation and phase retardance of the fore-optics set in the orbital environment.

To intuitively display the superposition state of the modulation spectrum in the optical path difference domain, we express it in the form of phase factor accumulation, as follows [16]:

(4) $$\begin{array}{ll} B_{orbit}= & X_{0}+b_{1,orbit}c_{1,orbit}X_{12}\\ & +\frac{1}{2}b_{2,orbit}c_{2,orbit}X_{12}\left[\exp\left(-i\delta_{2,orbit}\right)+\exp\left(i\delta_{2,orbit}\right)\right]\\ & -\frac{1}{4}b_{2,orbit}\left(1+c_{1,orbit}\right)X_{123}^{*}\exp\left[-i\left(\delta_{1,orbit}-\delta_{2,orbit}\right)\right]\\ & -\frac{1}{4}b_{2,orbit}\left(1+c_{1,orbit}\right)X_{123}\exp\left[i\left(\delta_{1,orbit}-\delta_{2,orbit}\right)\right]\\ & +\frac{1}{2}b_{1,orbit}c_{2,orbit}X_{123}^{*}\exp\left(-i\delta_{1,orbit}\right)\\ & +\frac{1}{2}b_{1,orbit}c_{2,orbit}X_{123}\exp\left(i\delta_{1,orbit}\right)\\ & +\frac{1}{4}b_{2,orbit}\left(1-c_{1,orbit}\right)X_{123}^{*}\exp\left[-i\left(\delta_{1,orbit}+\delta_{2,orbit}\right)\right]\\ & +\frac{1}{4}b_{2,orbit}\left(1-c_{1,orbit}\right)X_{123}\exp\left[i\left(\delta_{1,orbit}+\delta_{2,orbit}\right)\right], \end{array}$$

where $\delta_{1,orbit}$ and $\delta_{2,orbit}$ are the ideal retardances of $R_{1}$ and $R_{2}$ and the nine phase factors correspond to the nine distinct channels of the modulation spectrum. The channels can be obtained through Fourier transform:

(5) $$\begin{array}{l} C_{0}=F\left(X_{0}+b_{1,orbit}c_{1,orbit}X_{12}\right)\\ C_{1}=F\left[\frac{1}{2}b_{2,orbit}c_{2,orbit}X_{12}\exp\left(-i\delta_{2,orbit}\right)\right]\\ C_{-1}=F\left[\frac{1}{2}b_{2,orbit}c_{2,orbit}X_{12}\exp\left(i\delta_{2,orbit}\right)\right]\\ C_{2}=F\left\{-\frac{1}{4}b_{2,orbit}\left(1+c_{1,orbit}\right)X_{{}_{123}}^{*}\exp\left[-i\left(\delta_{1,orbit}-\delta_{2,orbit}\right)\right]\right\}\\ C_{-2}=F\left\{-\frac{1}{4}b_{2,orbit}\left(1+c_{1,orbit}\right)X_{123}\exp\left[i\left(\delta_{1,orbit}-\delta_{2,orbit}\right)\right]\right\}\\ C_{3}=F\left[\frac{1}{2}b_{1,orbit}c_{2,orbit}X_{123}^{*}\exp\left(-i\delta_{1,orbit}\right)\right]\\ C_{-3}=F\left[\frac{1}{2}b_{1,orbit}c_{2,orbit}X_{123}\exp\left(i\delta_{1,orbit}\right)\right]\\ C_{4}=F\left\{\frac{1}{4}b_{2,orbit}\left(1-c_{1,orbit}\right)X_{{}_{123}}^{*}\exp\left[-i\left(\delta_{1,orbit}+\delta_{2,orbit}\right)\right]\right\}\\ C_{-4}=F\left\{\frac{1}{4}b_{2,orbit}\left(1-c_{1,orbit}\right)X_{123}\exp\left[i\left(\delta_{1,orbit}+\delta_{2,orbit}\right)\right]\right\} \end{array}$$

It should be noted that $D_{fore-orbit}$ can be calibrated with the beams without a polarizer, as in the laboratory method, which is expressed as follows [16]:

(6) $$D_{fore-orbit}=\frac{2F^{-1}\left(G_{1}\right)}{a_{2,orbit}b_{2,orbit}c_{2,orbit}F^{-1}\left(G_{0}\right)\exp\left(-i\delta_{2,orbit}\right)-2a_{2,orbit}b_{1,orbit}c_{1,orbit}F^{-1}\left(G_{1}\right)}$$

where $G_{0}$ and $G_{1}$ are the corresponding channels with the beams without a polarizer. The water cloud zero degree of linear polarization (DOLP) calibration source [18,19] or the sun (usually regarded as lambertian) [13,20] are ideal calibration sources for obtaining the beams without a polarizer. $\varepsilon_{1}$, $\varepsilon_{2}$, $\delta_{1,orbit}$, and $\delta_{2,orbit}$ can be calibrated accurately by the method in Refs. [13,21]. The overall calibration scheme consists of multiple parameter calibrations, which are usually coupled. Based on the calibration sequence, these parameters, as the observed specific values, are used for numerical analysis of $\delta_{fore-orbit}$. The Stokes vector reconstruction model is expressed as [16]:

(7) $$\left\{\begin{array}{l} S_{0}=\frac{A_{0}-D_{fore}A_{1}}{1-D_{fore}^{2}},S_{1}=\frac{A_{1}-D_{fore}A_{0}}{1-D_{fore}^{2}}\\ S_{2}=\frac{A_{2}-\delta_{fore}A_{3}}{1+\delta_{fore}^{2}},S_{3}=\frac{A_{3}+\delta_{fore}A_{2}}{1+\delta_{fore}^{2}} \end{array}\right.$$

(8) A0=2F−1C0−2b1,orbitc1,orbitF−1C1b2,orbitc2,orbitexp−iδ2,orbitA1=a2,orbit2F−1C1b2,orbitc2,orbitexp−iδ2,orbit+a1,orbitRe4F−1C4b2,orbit1−c1,orbitexp−iδ1,orbit+δ2,orbitA2=a1,orbit2F−1C1b2,orbitc2,orbitexp−iδ2,orbit−a2,orbitRe4F−1C4b2,orbit1−c1,orbitexp−iδ1,orbit+δ2,orbitA3=−Im4F−1C4b2,orbit1−c1,orbitexp−iδ1,orbit+δ2,orbit

The overall calibration is based on the numerical operation of channel observations. Further, the construction sequence is unilinear. The channel observations and the calibrated parameters, can be used for subsequent analysis. Fortunately, we find a function to express the characteristic relationship between the AOP of the beam and the known quantity. For any partially linearly polarized beam $S_{in-orbit}=S_{0}{\left[1,P\sin2\theta ,P\cos2\theta ,0\right]}^{T}$ with a known degree of polarization (DOP) P, the function is written as:

(9) $$Z=\underset{\mathrm{Numerical}\;\mathrm{solution}}{\underset{︸}{\cos2\varepsilon_{1}\cos2\theta +\sin2\varepsilon_{1}\sin2\theta }}=\underset{\mathrm{Analytic}\;\mathrm{solution}}{\underset{︸}{\frac{1}{P}\left[\frac{\left(1-c_{1,orbit}\right)F^{-1}\left(C_{1}\right)F^{-1}\left(C_{3}\right)}{b_{2,orbit}c_{{}_{2,orbit}}^{2}S_{0}F^{-1}\left(C_{4}\right)}-a_{2,orbit}D_{fore-orbit}\right]}}$$

**Figure 2:**
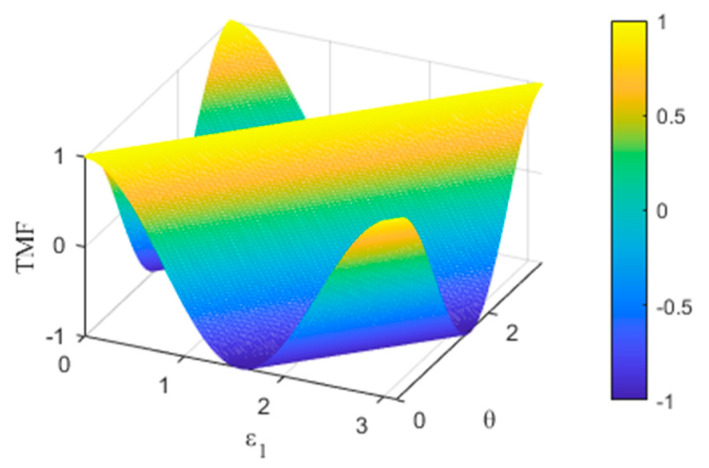
Numerical solution of TMF.

We name Z as Target-beam Monitoring Function (TMF). Figure 2 shows the numerical solution of TMF plotted within the unit value interval of the independent variables $\varepsilon_{1}$ and θ.

The only maximum condition of TMF is $\theta =\varepsilon_{1}$, excluding the impossibility of $\varepsilon_{1}=0^{\circ }$ (perfect installation) and $\varepsilon_{1}=180^{\circ }$ (reverse installation) in practical projects. Furthermore, the maximum is the specific value 1, which is direct feedback to “capture” the target beam with the specific AOP. Even though there are reasonable errors in the parameter calibrations, the acquisition of the beam with a specific AOP is not affected, which ensures the in-orbit calibration accuracy of the PROS.

To increase the calibration frequency, we choose the marine flares, common in orbit, as the calibration area. The flares, as ideal reflective media, have high radiation utilization. Moreover, according to Fresnel reflection, when the beam’s incident angle to the flares is greater than Brewster’s angle, the reflected beam is fully linearly polarized. Further, considering natural factors such as wind speed and foam, a more accurate rough sea surface model is required in practice. In the relevant research of many scholars, we select the anisotropic Breon and Henriot model (BHA) [22]. The BHA uses reflectance data over the global oceans taken by the Polarization and Directionality of the Earth’s Reflectances (POLDER) and wind data from NASA Scatterometer instruments. It has the highest correlation coefficient with the analytic DOP in the operating band. Assisted by the modified vector radiative transfer model and uncertainty analysis, the analytic DOP of the received polarized beam is fed back. The accuracy of the analytic DOP is controlled within 2%, meeting the calibration requirements. The above conditions endow the analytic solution of Z with instant parameters.

Using the “captured” reference beam, we obtain the in-orbit calibration value of $\delta_{fore}$ as follows:

(10) δfore−orbit=41+b1,orbitc1,orbit1+a2,orbitDfore−orbita1,orbitb2,orbit1−c1,orbit⋅ImF−1C4F−1C0exp−iδ1,orbit+δ2,orbit

## 3. Verification Process

### 3.1. Numerical Simulation 

In the numerical simulation, the subsystem is modularized based on the cascade rule of the Muller matrix. The Muller matrix of each subsystem can be obtained by polarization ray tracing. The operating band range, the high-order retarder’s thickness, the fore-optic’s structure, and the detector selection is referred to in Ref. [16]. The relevant data is used as the input value of the simulation. To strictly depict the integrity of Z, the tuning range of the DOP and the AOP of the simulated illuminant covers all possible conditions.

The demonstration of calibration in a continuous band is complex and abstract. Therefore, the center of the operating band is selected as the observation wavelength if necessary. In addition, the direct effect of pupil distribution on polarization aberration cannot be ignored. Therefore, according to the pupil distribution of $\delta_{fore}$, we choose the location with a high value (normalized field of view [0, 0.5]) as the observation point. $\varepsilon_{1}$ is set to 1°, 3°, 30°, and 45°. 

**Figure 3:**
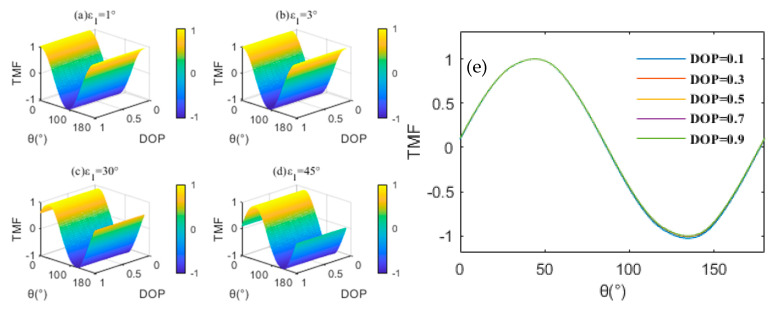
(**a**–**d**) Array of Z analytic values varying with the simulated illuminant. (**e**) Front view of array of Z analytic values at $\varepsilon_{1}{=45}^{{}^{\circ}}$.

**Figure 4:**
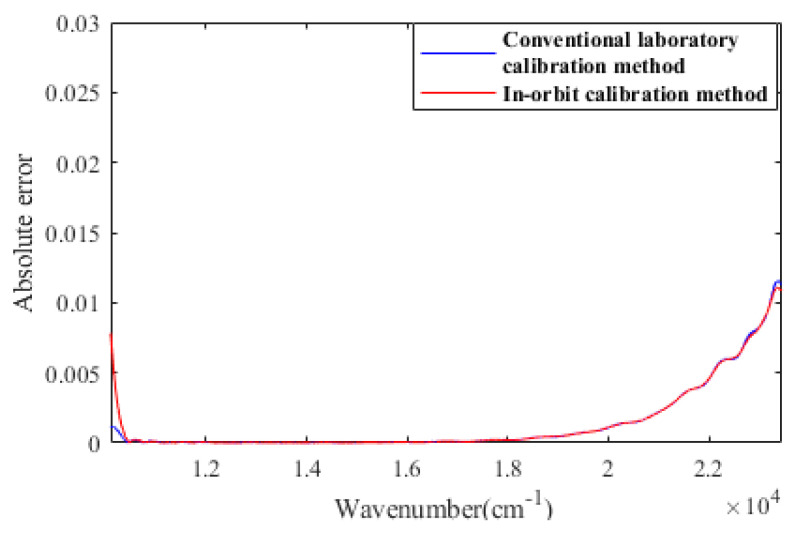
Calibration errors of $\delta_{fore}$ in simulated orbit and in laboratory.

The array of Z analytical values varying with the simulated illuminant is shown in Figure 3. In different cases, the analytical trend of Z is highly consistent with the calculation results based on the theoretical model. Furthermore, we reached some interesting conclusions: (a) The DOP is not coupled with other parameters as shown in Figure 3e. The error of the analytic DOP is not a factor affecting the analytical characteristics of Z; (b) Compared with Figure 3c,d, the maximum of Z in Figure 3a,b are too “dense” to be distinguished, which corresponds to $\varepsilon_{1}$ being considered as only caused by the alignment error. It proves that tiny $\varepsilon_{1}$ will directly impact the sensitivity and effectiveness of the TMF. It is necessary to consider making the angular difference between the actual setting position and the preset position of high-order retarders easier to distinguish. We compare the simulated in-orbit calibration method of $\delta_{fore}$ with the conventional laboratory calibration method at $\varepsilon_{1}=30{}^{\circ}$. Figure 4 compares calibration errors at 10,500–23,450 nm^−1^.

Due to the periodicity of the Fourier transform algorithm, the apodization function is used to suppress the ringing effect that may occur in the reconstruction. This processing inevitably leads to edge data exceptions. When this interference is eliminated, the calibration accuracy of $\delta_{fore-orbit}$ and $\delta_{fore-lab}$ are highly consistent.

### 3.2. Experimental Verification

**Figure 5:**
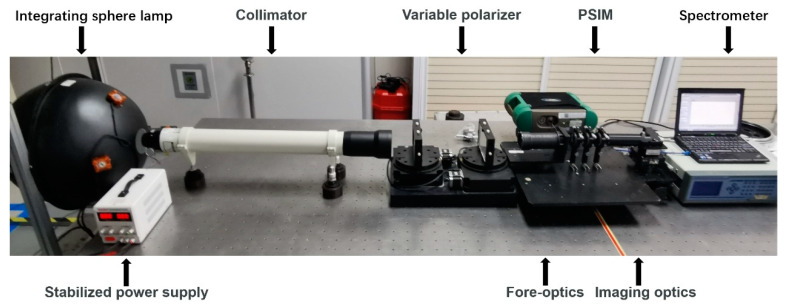
Photograph of the experimental configuration.

The instrument is in the pre-research stage. The approach to in-orbit test data is not yet available. Therefore, we built the configuration shown in Figure 5 for the calibration experiments. The functional unit consists of the PSIM and a spectrometer (Field Spec 3, Analytical Spectral Devices). The input data is consistent with the simulation.

The in-orbit scene is restored as much as possible. A variable polarizer is added to the illuminant-generating device. The polarization state of the target beam is obtained by Stokes polarimeter. In addition, possible ambient temperature disturbance in orbit is of consideration. The configuration is placed in the temperature control system. The regulating range is 20 °C to 30 °C, which is consistent with the temperature control requirements of the aircraft.

**Figure 6:**
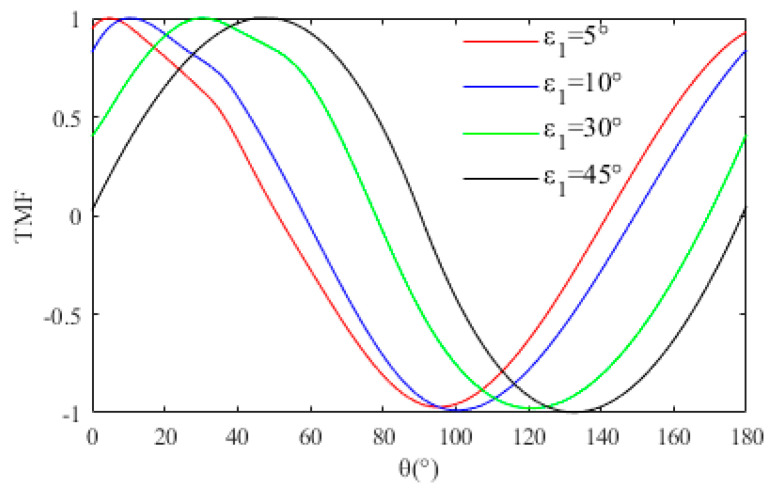
TMF response curve at P=1.

In the validation experiment of TMF, we set $\varepsilon_{1}$ and θ as adjusting variables. Based on the conclusion of the numerical simulation, Figure 6 shows the measurement results with a fully linearly polarized beam (P=1). When the polarization state of the target light changes continuously, the response distribution of TMF is consistent with the simulation results. In other words, TMF strictly has the only maximum condition “$\theta =\varepsilon_{1}$, Z=1”. The maximum is the specific value 1. The proposed method of “capturing” the target beam with a specific AOP is further verified.

**Figure 7:**
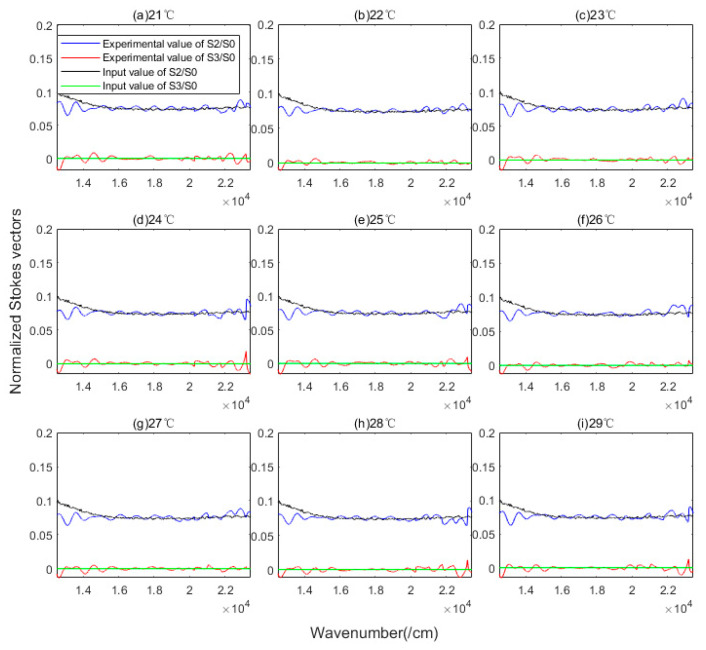
Normalized reconstructed $S_{2}/S_{0}$,$S_{3}/S_{0}$ at different temperatures.

In the multi-parameter coupling calibration scheme, it is not easy to highlight the influence of a single parameter. Therefore, we take the reconstruction accuracy of the Stokes vector as the traceability benchmark. The $\delta_{fore}$ is coupled to the reconstruction model of S2 and S3. Figure 7 compares the normalized reconstructed Stokes vector with the input value corresponding to different temperature settings. The reconstruction accuracy of S2 and S3 in the whole wavenumber domain by RMSE is 7.2 × 10^−3^ and 3.3 × 10^−3^.

The applicability of the scheme is analyzed as necessary. The scheme is not limited to adapting specific channeled spectropolarimeters or PROS in-orbit calibration. The grayscale value received by the spectrometer is added to the linear calibration operation as the only external input. It is ruled out that the polarization information of the polychromatic beam may cause crosstalk to channel construction. Therefore, for all the channeled spectropolarimeters characterized with the Muller matrix, TMF can be constructed similarly to accurately obtain beams with specific AOP. The beams with different AOPs can be obtained by adjusting the setting angle of high-order retarders as required. It provides a novel idea for constructing the overall in-orbit calibration scheme. It should be noted that a single reference beam is only valid in one calibration cycle. The effective way to improve the calibration accuracy is to increase the calibration frequency. The corresponding verification work will be supported by the in-orbit experimental data.

## 4. Conclusions

In sum, a novel scheme of PROS in-orbit calibration for the channeled spectropolarimeter is presented. To avoid the burden of the volume and design difficulty of the onboard calibrator, we build the Target-beam Monitoring Function to capture the beam with a specific AOP. In conjunction with the general derivation of the PROS, high-frequency calibration is realized. The numerical simulation and experiments prove the function’s validity and the high calibration precision. The highlight of the scheme is to simplify the calibration program and ensure that the PROS high-precision calibration is not disturbed by the orbital environment.

## Data Availability

The data presented in this study are available on request from the corresponding authors.
